# Associations between Cardiovascular Signal Entropy and Cognitive Performance over Eight Years

**DOI:** 10.3390/e23101337

**Published:** 2021-10-14

**Authors:** Silvin P. Knight, Louise Newman, Siobhan Scarlett, John D. O’Connor, James Davis, Celine De Looze, Rose Anne Kenny, Roman Romero-Ortuno

**Affiliations:** 1 The Irish Longitudinal Study on Ageing (TILDA), School of Medicine, Trinity College Dublin, D02 R590 Dublin, Ireland; louise.newman@tcd.ie (L.N.); siobhan.scarlett@tcd.ie (S.S.); john.oconnor@qub.ac.uk (J.D.O.); davisj5@tcd.ie (J.D.); deloozec@tcd.ie (C.D.L.); rkenny@tcd.ie (R.A.K.); romeroor@tcd.ie (R.R.-O.); 2 Discipline of Medical Gerontology, School of Medicine, Trinity College Dublin, D02 R590 Dublin, Ireland; 3 School of Medicine, Dentistry and Biomedical Sciences, The Patrick G Johnston Centre for Cancer Research, Queen’s University, Belfast BT9 7BL, UK; 4 Mercer’s Institute for Successful Ageing (MISA), St. James’s Hospital, D08 E191 Dublin, Ireland; 5 Global Brain Health Institute, Trinity College Dublin, D02 PN40 Dublin, Ireland

**Keywords:** sample entropy, cognition, cognitive decline, cardiovascular, blood pressure, TILDA

## Abstract

In this study, the relationship between non-invasively measured cardiovascular signal entropy and global cognitive performance was explored in a sample of community-dwelling older adults from The Irish Longitudinal Study on Ageing (TILDA), both cross-sectionally at baseline (*n* = 4525; mean (SD) age: 61.9 (8.4) years; 54.1% female) and longitudinally. We hypothesised that signal disorder in the cardiovascular system, as quantified by short-length signal entropy during rest, could provide a marker for cognitive function. Global cognitive function was assessed via Mini Mental State Examination (MMSE) across five longitudinal waves (8 year period; *n* = 4316; mean (SD) age: 61.9 (8.4) years; 54.4% female) and the Montreal Cognitive Assessment (MOCA) across two longitudinal waves (4 year period; *n* = 3600; mean (SD) age: 61.7 (8.2) years; 54.1% female). Blood pressure (BP) was continuously monitored during supine rest at baseline, and sample entropy values were calculated for one-minute and five-minute sections of this data, both for time-series data interpolated at 5 Hz and beat-to-beat data. Results revealed significant associations between BP signal entropy and cognitive performance, both cross-sectionally and longitudinally. Results also suggested that as regards associations with cognitive performance, the entropy analysis approach used herein potentially outperformed more traditional cardiovascular measures such as resting heart rate and heart rate variability. The quantification of entropy in short-length BP signals could provide a clinically useful marker of the cardiovascular dysregulations that potentially underlie cognitive decline.

## 1. Introduction

In recent decades, there has been a profound shift in ageing demographics and with this, there has been a corresponding increase in the prevalence of age-related diseases such as cognitive impairment and dementia [1]. Mild cognitive impairment (MCI) is defined as a decline in cognitive abilities that is worse than normative performance for a set age and education level, but does not meet the criteria for the diagnosis of dementia [2]. Reported prevalence of MCI varies widely, though some studies suggest that it could be as high as 42% in individuals over 65 years of age [3].

The concept of neurocardiovascular instability (NCVI) refers to abnormal neural control of the cardiovascular system, which may affect the dynamic behaviour of blood pressure (BP) and potentially alter end-organ structure and function [4]. Older individuals are more prone to NCVI due to age-related physiological changes in the cardiovascular system, cerebral blood flow, autonomic nervous system (ANS), and humoral system. The ANS is responsible for controlling the body’s visceral functions and maintaining homeostasis [5]. A key mechanism for regulating short-term control of systemic BP is the baroreceptor reflex; this involves the activation of stretch receptors in the walls of the carotid sinuses and aortic arch, sensory input from which travel via the cranial nerves IX, X, and the carotid sinus nerve to the brainstem, and onwards to the hypothalamus, cerebellum, substantia nigra, and cerebral hemispheres [4]. NCVI could increase the risk of cognitive impairment and dementia through associated alterations in cerebral blood flow, potentially deriving from impaired BP control. The brain is highly metabolically active, and precise regulation of cerebral blood flow is essential for maintaining a reliable and adequate supply of oxygen and nutrients to the brain, due to the limited substrate storage and high metabolic demand of this vital organ [6]. Understanding the mechanism of NCVI and its potential causal association with cognitive decline could help in the early detection of MCI and dementia, as well as support the development of preventative and therapeutic strategies to manage this poorly understood and burdensome disease [4]. We hypothesised that abnormalities in these physiological control mechanisms may be detectable and quantifiable by the level of disorder in short-length continuously measured BP signals.

Disorder in physiological signals can be assessed by means of entropy [7]. Entropy is a measure of irregularity/unpredictability, assigning lower entropy values to periodic, predictable data, and higher entropy values to irregular, unpredictable data. Multiple implementations of entropy have been proposed for the analysis of time-varying physiological signals including approximate entropy (ApEn), sample entropy (SampEn), multi-scale entropy, and cross entropy [8,9,10,11,12]. In 2000, Richman and Moorman [11] introduced SampEn. Briefly, given a time series of length *N*, SampEn is defined as the negative natural logarithm of the conditional probability that two trajectories of length *m* remain similar for *m* + 1, within a tolerance specified as ±r× standard deviation (SD) of the time series. Self-matches are not considered in the probability calculation for SampEn, unlike the also widely used ApEn [13]. Additionally, it has been demonstrated that SampEn is largely independent of the data length and can potentially provide more consistent results than ApEn [11].

In this study, we explored two approaches to SampEn calculation for BP data, utilising both time-series data interpolated at 5 Hz (one- and five-minute epochs) and beat-to-beat data (five-minute epochs). We investigated the associations between these entropy measures and global cognitive performance, both cross-sectionally at baseline and longitudinally over a four- to eight-year period in a large sample of community-dwelling older adults in Ireland.

## 2. Materials and Methods 

### 2.1. Study Population

This research was carried out as part of The Irish Longitudinal Study on Ageing (TILDA), an ongoing nationally representative prospective cohort study of community-dwelling adults [14,15]. TILDA collects information on the health, economic and social circumstances of people aged 50 and over in Ireland. Wave 1 of this study (baseline) took place between October 2009 and February 2011 (*n* = 8507), and subsequent data were collected approximately every 2 years over four longitudinal waves (wave 2: February 2012 to March 2013; wave 3: March 2014 to December 2015; wave 4: January to December 2016; wave 5: January to December 2018). Waves 1 and 3 included a comprehensive health assessment conducted at a dedicated health assessment centre; waves 2, 4 and 5 were non-health centre assessment waves. No individuals with a prior diagnosis of dementia or Alzheimer’s disease were recruited into this study at baseline (wave 1). The full cohort profile has been previously described in detail [14,15]. Ethical approval was granted for each wave from the Faculty of Health Sciences Research Ethics Committee at Trinity College Dublin, Dublin, Ireland, and all participants provided written informed consent. All research was performed in accordance with the Declaration of Helsinki.

### 2.2. Cardiovascular Measurements

At wave 1 health assessment, blood pressure waveforms were measured continuously at 200 Hz using a Finometer MIDI device (Finapres Medical Systems BV, Amsterdam, The Netherlands) and recorded via a 12-bit resolution analogue-to-digital converter. All measurements were carried out in a comfortably lit room, at an ambient temperature between 21 and 23 °C. Participants laid supine for five minutes and data from the last minute of supine rest (i.e., resting state: RS) was utilised for the main analyses presented in this study, in order to maximise data stationarity as much as possible. However, results are also presented for the full five minutes of RS data, both for the 5 Hz interpolated time-series and with beat-to-beat (BtB) series (i.e., not interpolated and successive beats evenly spaced) data. Signals for BtB systolic blood pressure (sBP) and diastolic blood pressure (dBP) were extracted using MATLAB (R2020b, The MathWorks, Inc., Natick, MA, USA). For the interpolated approach, M couples of beat-to-beat values [sBP(*i*), *T*(*i*)] were extracted, with *i* between 1 and M, and subsequently interpolated linearly between consecutive beats, from *T*(*i*) to *T*(*i* + 1), at 5 Hz to obtain *n* = 300 (one minute) and *n* = 1500 (five minutes) samples. Interpolated data were transformed using the method proposed by Tarvainen et al. to detrend and increase stationarity (*λ* = 10, cut-off frequency = 0.28 Hz). This method is based on a smoothness priors approach, and operates like a time-varying finite-impulse response high-pass filter [16]. Stationarity of the data was assessed, for both the original data and transformed one-minute data, via overall wide-sense stationarity (WSS), stationarity about the mean (or linear trend), and stationarity about the variance [17,18]. Of note, entropy values for the main results presented herein were not calculated on the BtB data with a prescribed number of beats; rather, the data were treated as one-minute time series, wherein the number of beats would vary per participant, mainly dictated by RS heart rate (HR). The reasoning behind this was that entropy, or level of unpredictability, in this section of data may be influenced by several important physiological mechanisms including RS HR, heart rate variability (HRV), and blood pressure variability (BPV). This idea was formally explored herein using data simulations. To calculate HR and HRV measures, a surface 3-lead electrocardiogram (ECG) was also continuously recorded at 4 kHz using a Medilog AR12 system (Schiller, Baar, Switzerland) during the same five-minute period of supine rest.

### 2.3. Entropy Analysis

Entropy analysis was performed in MATLAB using freely available code [19]. A detailed description of the algorithms used to compute SampEn has been previously reported in detail [11]; however, below we provide a brief overview.

$B_{i}^{m}\left(r\right)$ is defined as the number of template vectors $x_{m}\left(j\right)$ similar to $x_{m}\left(i\right)$ (within *r*) divided by *N* − *m* − 1, where *j* = 1…*N* − *m*, with *j* ≠ *i* (to avoid self-matches). The average $B_{i}^{m}\left(r\right)$ for all *i* is given as

(1) $$B^{m}\left(r\right)=\frac{1}{N-m}\overset{N-m}{\underset{i=1}{\sum }}B_{i}^{m}\left(r\right).$$

Similarly, we define $A_{i}^{m}\left(r\right)$ as the number of template vectors $x_{m+1}\left(j\right)$ similar to $x_{m+1}\left(i\right)$ (within *r*) divided by *N* − *m* − 1, where *j* = 1…*N* − *m*, with *j* ≠ *i*. The average $A_{i}^{m}\left(r\right)$ for all *i* is given as

(2) $$A^{m}\left(r\right)=\frac{1}{N-m}\overset{N-m}{\underset{i=1}{\sum }}A_{i}^{m}\left(r\right).$$

SampEn was then calculated as

(3) $$\mathrm{SampEn}\left(m,r,N\right)=-\ln\left(\frac{A^{m}\left(r\right)}{B^{m}\left(r\right)}\right).$$

In this study, *m* (embedding dimension; the length of the data segment being compared) was set to 2, as this has been shown to provide good statistical validity for SampEn measurements, especially for biological data [20,21]. An *r* (similarity criterion) of 0.15 was selected in line with previous recommendations for similar physiological data [22,23]. To assess the effects of data stationarity on entropy measures, SampEn was calculated for both the original and transformed one-minute data.

### 2.4. Heart Rate (HR) and Heart Rate Variability (HRV) Analysis

A band-pass filter and a proprietary algorithm were used to detect the R peak of each heart beat recorded on the ECG signal [24]. Linear interpolation was used to exclude supra-ventricular ectopic beats (detected using *Medilog Darwin* (Huntleigh Healthcare Ltd., Cardiff, UK) software) and noise from the signals. Time domain measures were derived from each five-minute epoch, namely the standard deviation of N-N (time between each normal heartbeat) intervals (SDNN [ms]), the square root of the mean squared difference of successive N-Ns (RMSSD), and the percentage of successive N-N intervals that differ by more than 50 ms (pNN50). Mean RS HR [beats-per-minute (bpm)] was also calculated for this full five-minute period. 

### 2.5. Assessment of Cognitive Function

Global cognitive function was assessed using the Mini Mental State Examination (MMSE) [25] at all five waves and the Montreal Cognitive Assessment (MOCA) [26] at waves 1 and 3. MOCA was measured at waves 1 and 3, and MMSE at wave 1, by a research nurse during an in-centre or at-home health assessment. MMSE was measured at waves 2, 3, 4, and 5 by a trained interviewer during an at-home interview. On completion of both assessments, participants were given a score from 0 to 30. Since both the MMSE and MOCA are subject to ceiling effects, as the majority of participants score 30, or close to 30, the numbers of errors were calculated with the outcome representing the count of errors made during the test (i.e., MMSE/MOCA score-30) and used in analyses rather than total score.

### 2.6. Covariates

As part of the TILDA assessment, the following self-reported measures were also recorded at each wave and were included as covariates in the fully adjusted models reported herein: age, sex, educational attainment, number of cardiovascular conditions (angina, high blood pressure, heart failure, heart murmur, abnormal heart rhythm, heart attack, high cholesterol), diabetes, alcohol consumption habits (CAGE) [27], smoking history, and cardiovascular medication use (coded using the Anatomical Therapeutic Chemical Classification (ATC): antihypertensive medications (ATC C02), diuretics (ATC C03), β-blockers (ATC C07), calcium channel blockers (ATC C08), and renin–angiotensin system agents (ATC C09)). Additionally, depressive symptoms were assessed using the Centre for Epidemiologic Studies Depression scale (CESD) [28] and physical activity was measured via the short form of the international physical activity questionnaire (IPAQ) [29]. Two seated systolic and diastolic blood pressure measurements were also obtained (separated by 1 min) using an OMRON digital automatic blood pressure monitor (Model M10-IT, Kyoto, Japan) and results are reported for the cohorts, though not controlled for in statistical models.

### 2.7. Statistical Analysis

Statistical analysis was performed using STATA 15.1 (StataCorp, College Station, TX, USA). Cross-sectional analysis was performed at baseline (wave 1) and mixed-effects models were used to examine the longitudinal associations (MMSE, waves 1 to 5; MOCA, waves 1 and 3). Due to the left-skewed distribution of the MMSE and MOCA error count data, Poisson regression models were employed in this study, with results reported as incident rate ratios (IRRs). An IRR ≥ 1 indicates worsening cognitive performance, i.e., an increase in the number of errors made for each unit increase in the measure of interest. Separate models were used to examine sBP and dBP to avoid collinearities. Two sets of models were used: (i) adjusted for age, sex, and education, and (ii) fully adjusted for all covariates defined previously. For the longitudinal analysis, all covariates were treated as time-varying fixed effects, with the exception of sex, which was treated as a time-invariant fixed effect. Longitudinal models were three-level with random intercepts to account for repeated measurements of participants, as well as measurements of participants within the same households. Age squared (age^2^) was also added to each model to account for a potential non-linear relationship between age and cognition [30]. Follow up waves were parameterised as a set of factor variables. Interaction terms with wave were included for all predictor variables in order to estimate cognitive performance at each wave, as well as the change across waves, while accounting for the changing/differing effects of covariates across waves. SDNN, RMSSD, and pNN50 variables were log-transformed prior to analysis due to the non-normally distributed nature of these data. In order to investigate how the more traditional cardiovascular measures (SDNN, RMSSD, pNN50, and RS HR), as well as SampEn calculated using a longer section of data (five minutes) and BtB approach, compared with sBP and dBP SampEn (calculated from one minute of interpolated 5 Hz data) in the fully adjusted models outlined above, standardised coefficients were calculated for these variables as

(4) $$\frac{X-\bar{X}}{\mathrm{SD}\left(X\right)}$$

where *X* is the measure of interest for a particular individual, $\bar{X}$ is the mean, and SD(*X*) is the standard deviation across the cohort. This allowed for the assessment of the effect sizes relative to the study cohort distribution. Statistical significance was set at *p* ≤ 0.05.

### 2.8. Sensitivity Analysis

Two separate sensitivity analyses were also conducted. The first repeated the full analyses on a subsample of participants aged ≥60 years at baseline, to assess whether results were predominantly driven by age. The second repeated the analyses excluding participants with low cognitive scores at baseline (MOCA < 20; MMSE < 24) [30,31], as well as self-reported history of Parkinson’s disease, stroke, or transient ischemic attack (TIA) at any of the waves included in the analyses; this was done to assess associations in the absence of baseline cognitive impairment and potentially confounding neurological disorders. 

### 2.9. Data Simulations

In order to investigate the potential influence of RS HR and HRV on the entropy measures, code was developed in MATLAB to simulate control data with Gaussian distribution, based on ranges of HR and HRV values. First, a vector was generated giving the BP value at each ‘beat’ (*y*-axis), *V_BP_*, using

(5) $$V_{BP}\left(i\right)=BPV\times N\left(\mu ,\sigma^{2}\right),$$

where *BPV* is the average SD of sBP measures from the present study (19.5 mmHg) and *N*(*μ*,*σ*^2^) is a random number generator. Next, a second vector was generated to provide the time spacings between ‘beats’ (*x*-axis), *V_TIME_*, given as

(6) $$V_{TIME}\left(i\right)=\frac{12000}{HR}+\left(\frac{HRV}{5}\times N\left(\mu ,\sigma^{2}\right)\right)+V_{TIME}\left(i-1\right),$$

for *i* > 1 (*V_TIME_*(1) = 0). The time axis along which the time spacing (Equation (6)) is calculated is discretised to intervals *dt* = 0.005 s (200 Hz). The samples from Equation (5) are positioned according to the time spacing (Equation (6)) and interpolated via the built-in MATLAB function *interp1* to provide simulated data at 200 Hz. These data were then truncated to 60 s and decimated to 5 Hz, providing data analogous to the real-world data in this study. The simulated data have Gaussian distribution (Equation (5)), and the time that separates data have Gaussian distribution (Equation (6)), with mean and variance that correspond to real-world data (HR (40–120 bpm) and HRV (1.8–165 ms)). SampEn was then calculated for each combination of HR and HRV, using the same input SampEn parameters as the main analyses (*m* = 2, *r* = 0.15, and *N* = 300), and the results were presented as a heat map plot. Heat map plots were also produced based on the real-world sBP and dBP data in order to allow for comparison between the SampEn results for the simulated and the real-world data. Since in a real-world scenario, it is likely that certain combinations of HR and HRV would be more common within the cohort than others, a heat map showing the number of participants within each group was also produced.

## 3. Results

### 3.1. Participant Characteristics

**Figure 1:**
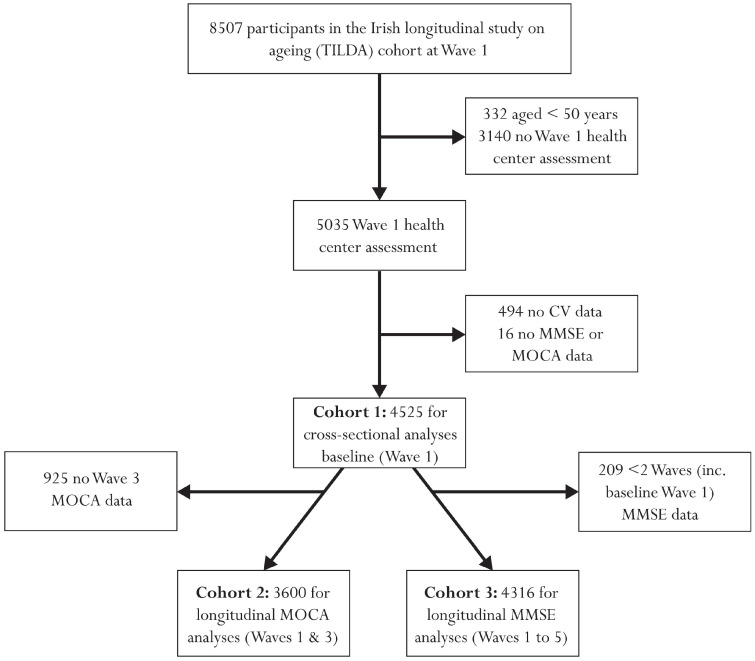
Flow chart describing sample selection and exclusions.

In total, 8507 participants were recruited at wave 1 of TILDA, of whom 5035 aged over 50 years attended for a health centre assessment at wave 1 (baseline). Adequate cardiovascular data were available for 4541 individuals for the calculation of baseline SampEn values, of whom wave 1 MOCA and MMSE data were also available for 4525; this cohort (Cohort 1: mean (SD) age: 61.9 (8.4) years; 54.1% female) was used for cross-sectional analysis of both MOCA and MMSE performance. Of these individuals, 3600 also had MOCA data at wave 3; this formed the second cohort (Cohort 2: mean (SD) age: 61.7 (8.2) years; 54.1% female), used for longitudinal analysis of MOCA performance. 4316 individuals had MMSE data for at least two of the five waves (including baseline wave 1; 3145 (72.9%) participants had data for all five waves, 502 (11.6%) for four waves only, 330 (7.6%) for three waves only, and 339 (7.9%) for two waves only), forming the third cohort (Cohort 3: mean (SD) age: 61.9 (8.4) years; 54.4% female), for longitudinal MMSE analysis. Full exclusions are illustrated in Figure 1. Across all three cohorts, 20–22% had primary education or less, 41–42% had secondary level education and 38–39% had attained a tertiary level education or higher. Full demographics for all three cohorts are provided in Table 1.

### 3.2. Associations of Entropy with Cognitive Performance

**Figure 2:**
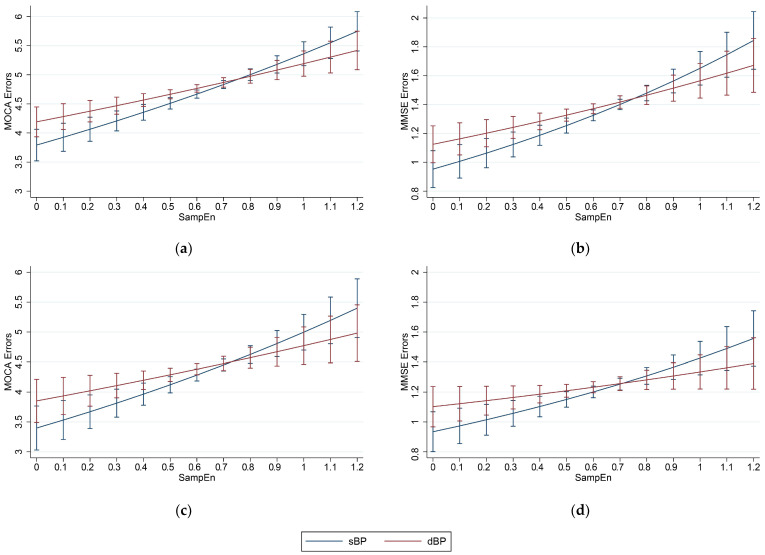
Marginal plots for cross-sectional models of (**a**) MOCA errors and (**b**) MMSE errors and longitudinal models of (**c**) MOCA errors and (**d**) MMSE errors, versus baseline (wave 1) systolic blood pressure (sBP) and diastolic blood pressure (dBP), sample entropy (SampEn; 1 min 5 Hz) measures. Models controlled for all covariates listed in Methods.

Results from adjusted regression analyses examining the associations between baseline SampEn and cognitive function are presented in Table 2 and Table 3, and illustrated in Figure 2 as margin plots. In cross-sectional models adjusted for age, sex, and educational attainment (Table 2), higher SampEn in sBP and dBP signals was associated with more errors in both MOCA and MMSE; similar results were found for fully adjusted cross-sectional models (Table 3). In the longitudinal models, higher SampEn in sBP and dBP measures were associated with more errors in both the MOCA and MMSE at all waves, for both age-, sex-, and education-adjusted and fully controlled models (Table 2 and Table 3). The direction of the associations remained constant across waves, i.e., higher sBP and dBP SampEn values were associated with more MOCA/MMSE errors. Based on absolute IRR values, sBP SampEn seemed more strongly associated with cognitive performance than dBP SampEn; however, the 95% CIs for IRRs generally overlapped (shown in Figure 2).

For the first sensitivity analysis (i.e., only participants aged ≥60 years), 2482, 1963, and 2363 individuals were available for cross-sectional, longitudinal MOCA, and longitudinal MMSE, respectively. For this analysis, all significant cross-sectional associations between SampEn and MOCA/MMSE errors were preserved. Likewise, longitudinal associations between SampEn and MMSE errors were also preserved. For longitudinal MOCA errors, the association with sBP SampEn was preserved. Results for the first sensitivity analyses are presented in Appendix A, Table A1. 

Results from the second sensitivity analysis (i.e., exclusions due to lower baseline cognitive performance and/or neurological disorders) are presented in Appendix A, Table A2. For cross-sectional analyses, 4128 participants’ data were available (246 excluded due to MOCA score < 20, 27 due to MMSE score < 24, 48 due to stroke, 9 due to Parkinson’s disease, and 67 due to TIA). Of the 3600 individuals included for longitudinal MOCA analyses, 172 were excluded due to lower baseline cognitive performance (MOCA score < 20, *n* = 154; MMSE score < 24, *n* = 18) to provide a non-cognitively impaired sample at baseline. A further 142 were excluded due to self-reported neurological disorders (waves 1–3: 44 due to stroke, 89 due to TIA, and 9 due to Parkinson’s disease), providing 3286 participants for the longitudinal MOCA sensitivity analyses. Of the 4316 individuals included for longitudinal MMSE analyses, a further 247 were excluded due to poor baseline cognitive performance (MOCA score < 20, *n* = 223; MMSE score < 24, *n* = 24). An additional 266 were excluded due to self-reported neurological disorders (waves 1–5: 86 due to stroke, 160 due to TIA, and 20 due to Parkinson’s disease), leaving 3803 individuals for inclusion in the longitudinal MMSE sensitivity analyses. For this second sensitivity analyses, all cross-sectional associations between SampEn and MOCA/MMSE errors remained significant. For longitudinal sensitivity analyses, the association between sBP SampEn and MOCA/MMSE errors were preserved.

Results from the stationarity tests (presented in Appendix B, Table A3) revealed low proportions of overall WSS (2.3% to 3.5%) for the untransformed data; overall WSS increased after transforming the data (4.8% to 6.4%). In the untransformed data, 48.5% to 50.6% of participants’ data were stationary about the mean, increasing to 84.7% to 91.3% of participants’ data in the transformed data. Similarly, 30.9% to 35.4% of participants’ data were stationary about the variance in the untransformed data, increasing to 43.1% to 54.9% for the transformed data. SampEn values calculated for the transformed data were on average 0.167 and 0.211 higher than the untransformed data, for sBP and dBP, respectively. All results presented herein were calculated using the transformed data; however, we also present results for the main analyses performed using the original, untransformed data in Appendix B, Table A4; these results did not differ widely from those obtained using the transformed data.

**Figure 3:**
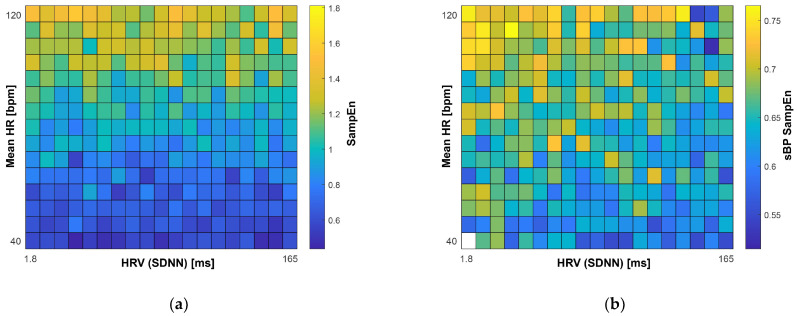

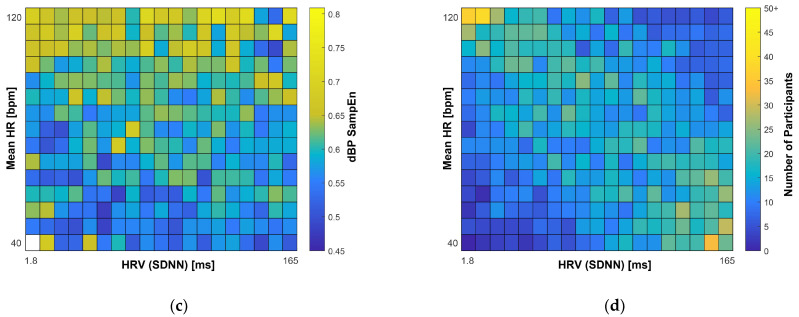
Heat map plots of (**a**) simulated sample entropy (SampEn) data and real-world (**b**) systolic (sBP) and (**c**) diastolic (dBP) blood pressure mean SampEn data (1 min 5 Hz) by groupings based on mean heart rate (HR) and heart rate variability (HRV). Density of participant within each group is also presented in (**d**). Groups for real-world data are composed of percentile groups, with overall minimum and maximum values shown in plots. Abbreviations: SDNN: standard deviation of N-N interval (time between each ‘normal’ heartbeat).

**Figure A1:**
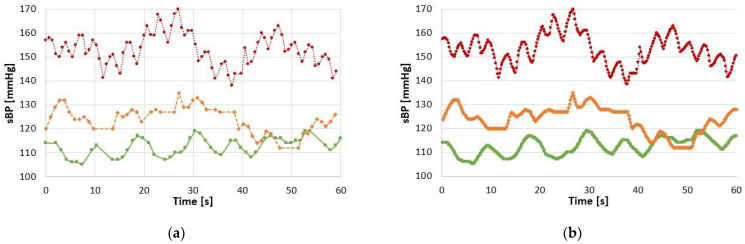

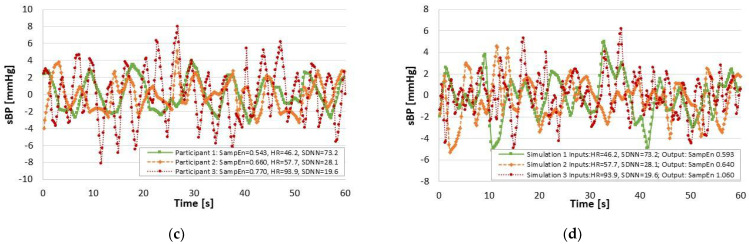
Example plots showing (**a**) beat-to-beat data, (**b**) data interpolated at 5 Hz from beat-to-beat data, (**c**) interpolated data transformed to make stationary, and (**d**) data simulated based on input parameters taken from example participant data heart rate (HR) and standard deviation of N-N interval (SDNN). Note, legend provided in (**c**) also applies to participant’s data shown in (**a**) and (**b**).

The results of the computer simulations that were designed to probe the relationship between BP SampEn and more traditional cardiovascular measures (RS HR and HRV (SDNN)) are presented as heat map plots in Figure 3. A trend was observed for SampEn to increase as RS HR was increased, and to a much lesser extent, a trend for SampEn values to decrease as HRV was increased, particularly at lower RS HR values (see Figure 3a). Similar trends were observed in the real-world sBP and dBP heat map plots, with a more apparent trend related to HRV in this instance (see Figure 3b,c). As shown in Figure 3d, in the real-world scenario, few participants had either the combinations of low RS HR and low HRV, or high RS HR and high HRV; indeed, the majority of participants with low HRV had high RS HR, and conversely those with high HRV had low RS HR. Simulated data provided higher SampEn values overall, compared with the real-world data (see Figure 3). Figure A1 (Appendix C) illustrates three example participants’ data, showing the (a) BtB data, (b) data interpolated at 5 Hz, and (c) the same data then transformed to increase stationarity. Figure A1 also shows (d) three examples of simulated data, generated using the code described in Section 2.9, with input HR and SDNN values from the three example participants.

Results from the fully adjusted regression analyses performed using standardised coefficients, which allowed for a comparison of the performance (in relation to associations with MMSE and MOCA errors) of more traditional cardiovascular measures (RS HR and HRV) and sBP/dBP SampEn, are presented in Table 4. For cross-sectional associations, higher SDNN and RMSSD values were associated with less errors in both MOCA and MMSE assessments (i.e., IRRs < 1). Conversely, higher mean RS HR was associated more errors in both MOCA and MMSE tests. As reported above, higher sBP and dBP SampEn in the one-minute data section were both associated with poorer performance on both MOCA and MMSE tests cross-sectionally, with both measures providing higher absolute IRRs when compared with RS HR, although their 95% CIs overlapped. For cross-sectional MMSE errors, sBP SampEn seemed to provide a larger effect size when compared with that of SDNN and RMSSD. In longitudinal analyses, RS HR was not significantly associated with cognitive performance. Higher SDNN was associated with better cognitive performance longitudinally, in both MOCA and MMSE assessments. Higher RMSSD was associated with better performance in the MOCA test longitudinally. As was the case with cross-sectional analyses, sBP SampEn (calculated from 5 Hz data) also seemed to provide a larger effect size, when compared with both SDNN and RMSSD, for longitudinal MMSE errors. No significant associations were found between pNN50 and cognitive performance.

Table 4 also reports results from the fully adjusted regression analyses performed using SampEn values calculated from five minutes of data, both linearly interpolated at 5 Hz and BtB, again reporting standardised coefficients to allow for comparison between models. For the interpolated approach, SampEn calculated from one minute of data provided similar results to values calculated using five minutes of data, in relation to cognitive performance. However, dBP SampEn calculated from five minutes of interpolated data was not significantly associated with longitudinal MOCA performance, though SampEn calculated from the one-minute section was. SampEn calculated using the BtB approach was not associated with cognitive performance, either cross-sectionally or longitudinally.

## 4. Discussion

In the present study, we utilised SampEn for the analysis of BP signal complexity and investigated the associations between these entropy measures and global cognitive performance, both cross-sectionally at baseline and longitudinally over a four-to-eight-year period in a large sample of community-dwelling older adults in Ireland. We found negative associations between BP SampEn measurements and global cognitive performance, after adjusting for multiple potential confounders. Cross-sectionally at baseline, higher sBP and dBP SampEn were associated with a greater number of MOCA and MMSE errors. In longitudinal models, higher SampEn, in both baseline sBP and dBP signals, was also associated with worse global cognitive performance at each wave. Results also suggested that the approach used herein for the calculation of entropy in short-length (60 s) BP data may be more strongly associated with cognitive performance, both cross-sectionally and longitudinally, than other widely and traditionally used cardiovascular measures (RS HR and HRV), for which longer data lengths have been recommended (≥5 min) for robust estimation [32]. 

In a previous study, we demonstrated that cardiovascular signal entropy calculated in this way (i.e., with data treated as a one-minute ‘time series’, rather than in a specific BtB manner) had significant associations with pre-disability frailty status in TILDA [20]. In the present study, we investigated this approach to BP signal entropy measurement with cognitive performance; to our knowledge, this has been conducted for the first time, which precludes the direct comparison of these results with previous works. However, we also demonstrated that entropy values derived in this way were influenced by both RS HR and HRV, and several previous studies have investigated these measures in the context of cognitive performance. The majority of these previous works have reported lower RS HR and higher HRV to be associated with better cognitive performance [4,33,34,35,36,37].

In a 2000 study, Kennedy and Scholey found that individuals with baseline HR below the median of their cohort performed better on both Serial Threes and Serial Sevens cognitive assessments [33]. In a study of patients post-ischemic stroke, Böhm et al. reported low RS HR to be associated with less cognitive decline [34]. In another similar study of 54 patients with first-ever ischemic stroke, Tessier et al. found that lower HR and higher HRV were predictive of better global cognitive function at 3-month follow-up [35]. Other previous studies have also investigated the associations between HRV (as also assessed by traditional measures, such as SD of all R–R intervals or the root mean square of successive SD) and cognitive performance, with higher HRV reported to be associated with better cognitive performance [36,37]. It is thought that traditional HRV measures may be reflective of the overall flexibility of the cardiovascular system, i.e., the ability of the system to adapt to multiple challenges over a longer time period, a measure that is known to be indicative of a more positive health status [38]. Consistently, in the current study, the associations between traditional RS HR and HRV measures and cognitive performance were also found to be in the same directions, i.e., lower RS HR (cross-sectionally) and higher HRV (cross-sectionally and longitudinally) being associated with better cognitive performance (i.e., less MOCA and MMSE errors).

Both RS HR and SDNN were related to SampEn values (as illustrated in Figure 3). Indeed, higher RS HR was related to higher SampEn values, and conversely, those with higher HRV values tended to have lower BP SampEn. These observations (i.e., that BP SampEn calculated using 5 Hz interpolated time-series data appears to provide a composite measure of different important physiological factors) may partially explain the strong associations found between BP SampEn and cognitive performance, when considered in the context of previous HR/HRV studies. We postulate that other physiologically important features, such as BPV, would also exsert a strong influence on BP SampEn values calculated in this way. Indeed, previous work has suggested that complexity indexes assessed for HR and sBP provide complementary information, which can be used for the detection of early abnormalities in both cardiac and vascular controls [39]. Also of note, when comparing the results from the current study with previous works, are the large differences with the time scales investigated across studies; the timescale at which complexity is examined within a biological system most likely reflects different physiological processes and impairments in the coupling between physiological systems [39,40]. 

We suggest that entropy calculated in short-length BP data, via the methodologies reported herein, may be thought of as a measure of overall systemic disorder, or ‘jitter’, resulting from dysregulation of the cardiovascular system, as has been previously defined by NCVI [4]. Several simultaneously active regulatory mechanisms are responsible for short-term cardiovascular control [39,41]. One possible physiological cause for this dysregulation could be abnormally modified baroreflex sensitivity and/or vagal tone, since vagal activity has been previously demonstrated to be associated with the non-linear dynamics of heart period complexity [39,42]. Another plausible reason for this dysregulation could be an increase in sympathetic activity and/or modulation directed to the heart and/or blood vessels, as previously described in pathological ageing states [43]. Other potential influencing factors may include modified cardiac reserve, changes in arterial structure (e.g., increased stiffness, decreased compliance (i.e., decreased buffering to allow local flow oscillations), and endothelial dysfunction), as well as changes of diastolic filling and increased collagen in the left ventricle [44,45]. An adequate and consistent supply of blood to the brain is imperative for maintaining good cognitive function. Dysregulation of the cardiovascular system, through the mechanisms described above, can impair or interrupt cerebral blood flow, potentially leading to worse cognitive performance over time [4,46,47]. In fact, previous work has reported that the presence of NCVI at least doubles conversion rates from MCI to dementia, and that patients with MCI have a higher prevalence of NCVI and autonomic dysfunction than controls [48]. Results from the present study support the link between dysregulation of the cardiovascular system and reduced cognitive performance. Further work will be required to fully elucidate the origins of this potential measure of cardiovascular dysregulation, keeping in mind that this is most likely driven by a composite of the above potential influencing factors, as well as possibly other physiological and non-physiological factors. Since (to the authors’ knowledge) this is only the second study to use this approach to entropy calculation (i.e., using short-length interpolated BP data), replication of the methods utilised herein with other datasets/outcomes would also be of benefit to the field, to ensure the robustness of this approach.

Also worth noting with regards the main entropy approach used in the present work, despite the fact that the datapoints between successive beats were linearly interpolated, and therefore not technically a direct physiological measure, the slopes of these ‘between-beats’ data sections are directly related to the positions of the sBP and dBP BtB data points in the time series (i.e., BP/BPV dictates the y-axis position and HR/HRV the x-axis position of each ‘beat’), and therefore indirectly contain physiologically important information. One limitation to calculating entropy using linearly interpolated data is that the interpolation process inevitable decreases the irregularity of the series, in turn reducing the absolute SampEn values, compared with a BtB approach to entropy calculation. This was evident in the results from the BtB analyses reported herein, which provided mean SampEn values more than double those from the interpolated approach, as shown in Table 1. This is in line with previous cardiovascular entropy studies, which have reported mean SampEn values calculated in BtB data of >1.2; compared with mean sBP SampEn ~1.67 in the present study [23,49,50]. Despite this limitation, SampEn values calculated from both one and five minutes of 5 Hz interpolated data were significantly associated with cognitive performance, whereas SampEn values calculated from the BtB data were not. We hypothesise that this might be due to the fact that using the interpolated approach, both BP magnitude and the temporal spacing of successive beats are taken into account, as discussed above. Also of note is that SampEn calculated from either one or five minutes of interpolated data resulted in similar associations with cognitive performance, both cross-sectionally and longitudinally, implying that the use of one minute of resting-state data may be adequate for this approach. However, an important caveat to this approach is that there is most certainly a lower limit to the minimum length of data required for SampEn values calculated from linearly interpolated cardiovascular data to still remain a valid physiological measure, since very short data lengths (<1 min) may not contain a sufficient number of beats for robust entropy calculations; investigating this lower limit further in future work would be of benefit. There are many different signal processing approaches that can be taken with these type of time-varying data prior to SampEn calculation, such as filtering [51], down-sampling [52], and differencing [53], all of which have been shown to impact SampEn values [53,54]; future work replicating the techniques proposed in the present study should help to develop an optimal approach to the calculation of SampEn in this type of continuously measured BP data, based on how these processing techniques impact the sensitivity of SampEn to detect clinically meaningful health outcomes, such as cognitive performance in the case of this study. Indeed, even though there are many signal processing approaches which can be used for the calculation of entropy in cardiovascular data, the extent to which they compare in their correlation with clinical outcomes has been much less investigated [54]. SampEn was higher overall in the simulated control data, compared with the real-world data, and this may be due to several factors. In the simulation, each timepoint was treated as independent (i.e., not influenced by preceding of subsequent datapoints), and was allowed to vary as such; this is not the case with real-world physiological data, as multiple other homeostatic mechanisms are at play within the body, constantly attempting to moderate BP. The simulation did not model any overall trends across the generated datasets, and these trends, we propose, may have resulted in lower entropy values [55]. Additionally, the simulated data were produced using the assumption of a Gaussian distribution, for both the time and BP vectors, which is not the case with real-world data; this also may have contributed to discrepancies between real-world and simulated data SampEn absolute values.

Sensitivity analysis showed similar results to the main analysis, with regards associations between BP SampEn and MOCA/MMSE errors, even with a more aged sub-cohort (≥60 years), implying that the associations reported in this study are not primarily driven by age. Further sensitivity analysis also revealed that even when mildly cognitively impaired individuals were excluded at baseline, and those with neurological disorders were excluded at each wave, worse cognitive performance was still associated with higher baseline entropy in BP signals. This reinforces the value of non-invasively measured cardiovascular entropy as a potential early marker of cognitive decline. Future longitudinal work investigating how cardiovascular entropy measures vary over time would also be of interest, since tracking this measure in individuals may provide an ‘early warning’ for the onset of MCI or dementia, for possible application in clinical settings or population screening programmes. Future population-based studies at longer follow-up periods and/or in real clinical samples with participants at higher risk of more marked cognitive decline should clarify whether cognitive decline for higher entropy continues at a more rapid pace than lower entropy.

The present study has several strengths and other potential avenues for future work. The methodologies used herein were specifically designed to be highly transferable for use in a clinical setting. All measures were non-invasive and non-ionising. The short data length required (one minute), measured during supine rest, would be feasible and practical for use in a busy clinical setting, where the shorter time frame needed to collect the data would be a considerable advantage. Entropy provides a single-number measure derived directly from the time-series data with minimal pre-processing, which could theoretically be calculated at and displayed on the measurement device itself, allowing for easy use by clinicians. Input parameters and implementation of SampEn calculations were based on recommendations for similar physiological data from previous studies (*m* = 2 [7,21]; *r* = 0.15 [22,23]); however, a consensus with regards the optimal methodologies to use, as well as normative age- and sex-adjusted reference entropy values, would most likely be required for widespread clinical adoption of this technique. Further work is necessary to establish the prognostic implications of entropy measures vis à vis other clinical markers (e.g., for the prediction of mortality and other adverse health events). Another strength of this study is the large cohort sizes, as well as (in the case of MMSE) data being available at five time points, spanning an eight-year period.

There are also several additional limitations to this study that should be kept in mind when interpreting the results. In the current work, it was not possible to establish the directionality of the relationship between BP entropy and cognitive performance, i.e., is dysregulation of the cardiovascular system driving cognitive decline, or is a decline in cognition influencing sympathetic and/or parasympathetic cardiovascular control? This question should be the subject of future work. Data utilised in this study had relatively high proportions of non-stationarity, as is commonly the case with physiological data [56], which may have potentially biased the estimates of complexity since non-stationarities have been shown to diminish the absolute level of complexity as assessed by entropy [55]. However, efforts were made to ensure the data were as stationary as was feasible and practically possible, by using data from the last minute of supine rest and via transforming the data during pre-processing to remove trends. Future work exploring different methods to further increase stationarity of these types of data, while still retaining the physiologically relevant signal complexity information, would be of interest. In the present study, SampEn was used to investigate BP signal complexity at a single scale; future work using other entropy methods and approaches, such as multiscale, to the data could be of interest.

## 5. Conclusions

This study reported correlations between SampEn calculated from short-length BP data and global cognitive performance, both cross-sectionally at baseline, and longitudinally over an eight-year period. Though it was demonstrated that the novel approach taken to calculate entropy in these data was related to other commonly and traditionally used cardiovascular measures, namely RS HR and HRV, it was also demonstrated that SampEn calculated from one minute of interpolated RS BP data seemed to outperform these traditional measures with regards associations with cognitive performance. Further work to understand the physiological mechanisms underpinning these cardiovascular entropy measures, and their association with cognitive decline, could help in the detection and understanding of MCI and dementia, as well as support the development of preventative and therapeutic strategies to reduce the scale and impact of these growing conditions.

## Figures and Tables

**Table 1:** Demographic characteristics of the study samples.

	Cohort 1: Baseline Cross-Sectional (*n* = 4525)	Cohort 2: Longitudinal MOCA (*n* = 3600)	Cohort 3: Longitudinal MMSE (*n* = 4316)
**Age [years]**	61.9 (SD: 8.4, range: [50–91])	61.7 (SD: 8.2, range: [50–89])	61.9 (SD: 8.4, range: [50–91])
**Sex [% (*n*)]**	Female: 54.1% (2448)	Female: 54.1% (1947)	Female: 54.4% (2347)
**Education [% (*n*)]**			
Primary/None	21.5% (972)	19.8% (771)	21.1% (911)
Secondary	41.6% (1883)	41.3% (1488)	41.4% (1787)
Third/Higher	36.9% (1670)	38.9% (1401)	37.5% (1618)
**Physical Activity (IPAQ) [% (*n*)]**			
Low	27.5% (1244)	26.9% (969)	27.4% (1182)
Moderate	35.8% (1619)	35.7% (1285)	35.5% (1553)
High	36.0% (1628)	36.6% (1319)	36.3% (1567)
No response	0.8% (34)	0.8% (27)	0.8% (34)
**Self-Reported Diabetic [%]**	6.5% (293)	6.2% (224)	6.4% (277)
**Number of Cardiovascular Conditions [% (*n*)]**			
0	39.5% (1789)	39.5% (1423)	49.4% (1699)
1	34.9% (1577)	35.1% (1263)	34.9% (1508)
2+	25.6% (1159)	25.4% (614)	25.7% (1109)
**Antihypertensive Medication Use [% (*n*)]**	33.1% (1497)	32.4% (1166)	33.2% (1433)
**CAGE Alcohol Scale**			
CAGE < 2	78.1% (3535)	79.3% (2854)	78.4% (3384)
CAGE ≥ 2	12.9% (583)	13.3% (481)	13.1% (565)
No response	9.0% (407)	7.4% (265)	8.5% (367)
**Smoker [% (*n*)]**			
Never	45.8% (2074)	46.7% (1681)	46.3% (1996)
Past	39.3% (1776)	39.6% (1427)	39.2% (1693)
Current	14.9% (675)	13.7% (492)	14.5% (627)
**CESD [% (*n*)]**			
Non-depressed (CESD < 9)	86.2% (3902)	86.9% (3130)	86.3% (3726)
Depressed (CESD ≥ 9)	13.8% (623)	13.1% (470)	13.7% (590)
**Seated sBP [mmHg]**	134.5 (SD: 19.5, range: [78.5–220])	134.0 (SD: 19.4, range: [78.5–215])	134.3 (SD: 19.4, range: [78.5–220])
**Seated dBP [mmHg]**	82.3 (SD: 11.1, range: [51.5–132])	82.1 (SD: 11.1, range: [51.5–132])	82.2 (SD: 11.1, range: [51.5–132])
**SampEn sBP (1 min, 5 Hz)**	0.655 (SD: 0.125, range: [0.017–1.136])	0.652 (SD: 0.124, range: [0.017–1.065])	0.655 (SD: 0.124, range: [0.017–1.065])
**SampEn dBP (1 min, 5 Hz)**	0.597 (SD: 0.134, range: [0.019–1.281])	0.595 (SD: 0.131, range: [0.019–1.111])	0.597 (SD: 0.133, range: [0.019–1.140])
**SampEn sBP (5 min, 5 Hz)**	0.694 (SD: 0.106, range: [0.071–1.260])	0.692 (SD: 0.105, range: [0.071–1.185])	0.694 (SD: 0.106, range: [0.071–1.185])
**SampEn dBP (5 min, 5 Hz)**	0.640 (SD: 0.120, range: [0.069–1.239])	0.638 (SD: 0.118, range: [0.069–1.134])	0.640 (SD: 0.119, range: [0.069–1.134])
**SampEn sBP (5 min, BtB)**	1.672 (SD: 0.484, range: [0.339–3.957])	1.669 (SD: 0.480, range: [0.339–3.903])	1.673 (SD: 0.484, range: [0.339–3.957])
**SampEn dBP (5 min, BtB)**	1.444 (SD: 0.343, range: [0.405–3.460])	1.442 (SD: 0.344, range: [0.405–3.460])	1.445 (SD: 0.345, range: [0.405–3.460])

**Table 2:** Regression results for cross-sectional and longitudinal (mixed-effects multilevel regression) associations between baseline (wave 1) systolic blood pressure (sBP) and diastolic blood pressure (dBP) sample entropy (SampEn) measures (1 min 5 Hz) and MOCA/MMSE errors. Models controlled for age, sex, and educational attainment.

Cognitive Measure	CV Measure (SampEn)	IRR	*p*	95% CIs	*n*
MOCA Errors W1	sBP	1.46	**<0.001**	1.31 to 1.62	4525
	dBP	1.26	**<0.001**	1.15 to 1.40	4525
MMSE Errors W1	sBP	1.82	**<0.001**	1.49 to 2.22	4525
	dBP	1.43	**<0.001**	1.20 to 1.72	4525
MOCA Errors W1 and 3	sBP	1.45	**<0.001**	1.21 to 1.74	3600
	dBP	1.26	**0.010**	1.06 to 1.49	3600
MMSE Errors W1–5	sBP	1.81	**<0.001**	1.37 to 2.39	4316
	dBP	1.44	**0.005**	1.12 to 1.87	4316

Abbreviations: IRR: incident rate ratio; CIs: confidence intervals.

**Table 3:** Regression results for cross-sectional and longitudinal (mixed-effects multilevel regression) associations between baseline (wave 1) systolic blood pressure (sBP) and diastolic blood pressure (dBP) sample entropy (SampEn) measures (1 min 5 Hz) and MOCA/MMSE errors. Models controlled for all covariates listed in Methods.

Cognitive Measure	CV Measure (SampEn)	IRR	*p*	95% CIs	*n*
MOCA Errors W1	sBP	1.39	**<0.001**	1.25 to 1.55	4525
	dBP	1.23	**<0.001**	1.11 to 1.36	4525
MMSE Errors W1	sBP	1.69	**<0.001**	1.38 to 2.05	4525
	dBP	1.37	**0.001**	1.14 to 1.65	4525
MOCA Errors W1 and 3	sBP	1.43	**<0.001**	1.19 to 1.71	3600
	dBP	1.24	**0.017**	1.04 to 1.47	3600
MMSE Errors W1–5	sBP	1.80	**<0.001**	1.36 to 2.37	4316
	dBP	1.43	**0.007**	1.10 to 1.84	4316

Abbreviations: IRR: incident rate ratio; CIs: confidence intervals.

**Table 4:** Regression results for cross-sectional and longitudinal models of MOCA and MMSE errors versus baseline (wave 1) systolic (sBP SampEn) and diastolic (dBP SampEn) blood pressure sample entropy (calculated for one and five minutes of interpolated 5 Hz time-series data and five minutes beat-to-beat (BtB) data), heart rate variability (HRV; SDNN: standard deviation of N-N interval (time between each ‘normal’ heartbeat); RMSSD: square root of the mean squared difference of successive N-Ns; pNN50: percentage of successive N-N intervals that differ by more than 50 ms), and resting state heart rate (RS HR) measures. Models controlled for all covariates listed in Methods and all cardiovascular measures standardised to the cohort’s mean and standard deviation prior to analysis to allow for comparison between models.

Cognitive Measure	Cardiovascular Measure (Standardised-Per 1 SD)	IRR	*p*	95% CIs
MOCA Errors W1	sBP SampEn (1 min 5 Hz)	1.042	**<0.001**	1.029 to 1.056
	dBP SampEn (1 min 5 Hz)	1.028	**<0.001**	1.014 to 1.042
	sBP SampEn (5 min 5 Hz)	1.051	**<0.001**	1.037 to 1.065
	dBP SampEn (5 min 5 Hz)	1.021	**0.003**	1.007 to 1.034
	sBP SampEn (5 min BtB)	1.007	0.286	0.994 to 1.021
	dBP SampEn (5 min BtB)	1.001	0.925	0.988 to 1.014
	HRV log(SDNN)	0.943	**<0.001**	0.930 to 0.957
	HRV log(RMSSD)	0.970	**<0.001**	0.955 to 0.984
	HRV log(pNN50)	0.985	0.078	0.968 to 1.002
	Mean RS HR	1.024	**0.001**	1.010 to 1.039
MMSE Errors W1	sBP SampEn (1 min 5 Hz)	1.068	**<0.001**	1.041 to 1.094
	dBP SampEn (1 min 5 Hz)	1.043	**0.001**	1.018 to 1.069
	sBP SampEn (5 min 5 Hz)	1.071	**<0.001**	1.045 to 1.098
	dBP SampEn (5 min 5 Hz)	1.033	**0.008**	1.009 to 1.059
	sBP SampEn (5 min BtB)	1.000	0.981	0.976 to 1.026
	dBP SampEn (5 min BtB)	1.004	0.771	0.980 to 1.028
	HRV log(SDNN)	0.935	**<0.001**	0.910 to 0.959
	HRV log(RMSSD)	0.964	**0.005**	0.940 to 0.989
	HRV log(pNN50)	0.981	0.208	0.953 to 1.011
	Mean RS HR	1.033	**0.012**	1.007 to 1.060
MOCA Errors W1 and 3	sBP SampEn (1 min 5 Hz)	1.045	**<0.001**	1.022 to 1.069
	dBP SampEn (1 min 5 Hz)	1.028	**0.017**	1.005 to 1.052
	sBP SampEn (5 min 5 Hz)	1.054	**<0.001**	1.030 to 1.079
	dBP SampEn (5 min 5 Hz)	1.017	0.154	0.994 to 1.040
	sBP SampEn (5 min BtB)	1.016	0.170	0.993 to 1.040
	dBP SampEn (5 min BtB)	0.999	0.927	0.977 to 1.022
	HRV log(SDNN)	0.937	**<0.001**	0.915 to 0.961
	HRV log(RMSSD)	0.969	**0.011**	0.946 to 0.993
	HRV log(pNN50)	0.980	0.138	0.954 to 1.007
	Mean RS HR	1.023	0.072	0.998 to 1.047
MMSE Errors W1–5	sBP SampEn (1 min 5 Hz)	1.071	**<0.001**	1.036 to 1.107
	dBP SampEn (1 min 5 Hz)	1.047	**0.007**	1.012 to 1.082
	sBP SampEn (5 min 5 Hz)	1.078	**<0.001**	1.041 to 1.116
	dBP SampEn (5 min 5 Hz)	1.040	**0.024**	1.005 to 1.077
	sBP SampEn (5 min BtB)	1.002	0.906	0.968 to 1.038
	dBP SampEn (5 min BtB)	1.012	0.502	0.978 to 1.047
	HRV log(SDNN)	0.935	**<0.001**	0.901 to 0.970
	HRV log(RMSSD)	0.977	0.207	0.941 to 1.013
	HRV log(pNN50)	0.983	0.425	0.944 to 1.025
	Mean RS HR	1.033	0.074	0.997 to 1.070

Abbreviations: IRR: incident rate ratio; CIs: confidence intervals.

## Data Availability

The datasets generated during and/or analysed during the current study are not publicly available due to data protection regulations but are accessible at TILDA on reasonable request. The procedures to gain access to TILDA data are specified at https://tilda.tcd.ie/data/accessing-data/ (access on: 12 October 2021).

## Appendix A

**Table A1:** Regression results sensitivity analysis 1 (only ≥60 years of age included) for cross-sectional and longitudinal models of MOCA errors and MMSE errors versus baseline (wave 1) systolic blood pressure (sBP) and diastolic blood pressure (dBP) sample entropy (SampEn) measures. Models controlled for all covariates listed in Methods.

Cognitive Measure	CV Measure (SampEn)	IRR	*p*	95% CIs	*n*
MOCA Errors W1	sBP	1.33	**<0.001**	1.17 to 1.52	2482
	dBP	1.19	**0.005**	1.05 to 1.34	2482
MMSE Errors W1	sBP	1.65	**<0.001**	1.30 to 2.09	2482
	dBP	1.37	**0.005**	1.10 to 1.70	2482
MOCA Errors W1 and 3	sBP	1.29	**0.027**	1.03 to 1.62	1963
	dBP	1.18	0.124	0.96 to 1.46	1963
MMSE Errors W1–5	sBP	1.66	**0.003**	1.19 to 2.32	2363
	dBP	1.38	**0.044**	1.01 to 1.87	2363

Abbreviations: IRR: incident rate ratio; CIs: confidence intervals.

**Table A2:** Regression results sensitivity analysis 2 (baseline poor cognitive performance (MMSE < 24 or MOCA < 20), and/or history of Parkinson’s disease, TIA, and/or stroke excluded)) for cross-sectional and longitudinal models of MOCA errors and MMSE errors versus baseline (wave 1) systolic blood pressure (sBP) and diastolic blood pressure (dBP) sample entropy (SampEn) measures. Models controlled for all covariates listed in Methods.

Cognitive Measure	CV Measure (SampEn)	IRR	*p*	95% CIs	*n*
MOCA Errors W1	sBP	1.26	**<0.001**	1.12 to 1.42	4128
	dBP	1.15	**0.016**	1.03 to 1.28	4128
MMSE Errors W1	sBP	1.51	**<0.001**	1.20 to 1.91	4128
	dBP	1.24	**0.047**	1.00 to 1.54	4128
MOCA Errors W1 and 3	sBP	1.28	**0.010**	1.06 to 1.54	3286
	dBP	1.16	0.106	0.97 to 1.38	3286
MMSE Errors W1–5	sBP	1.56	**0.004**	1.16 to 2.10	3803
	dBP	1.25	0.117	0.95 to 1.67	3803

Abbreviations: IRR: incident rate ratio; CIs: confidence intervals.

## Appendix B

**Table A3:** Pre-processing results reporting the proportions of stationarity based on the wide-sense stationarity (WSS), mean stationarity, and variance stationarity tests, as well as differences in sample entropy (ΔSampEn) measures between the untransformed and transformed data.

Measure	Mean Stationary	Variance Stationary	Wide-Sense Stationarity (WSS)	ΔSampEn
**Untransformed Data**				
sBP	50.6%	35.4%	3.5%	
dBP	48.5%	30.9%	2.3%	
**Transformed Data**				
sBP	91.3%	54.9%	6.4%	0.167
dBP	84.7%	43.1%	4.8%	0.211

Abbreviations: sBP: systolic blood pressure; dBP: diastolic blood pressure.

**Table A4:** Regression results, using the raw, untransformed data, for cross-sectional and longitudinal models of MOCA errors and MMSE errors versus baseline (wave 1) systolic blood pressure (sBP) and diastolic blood pressure (dBP) sample entropy (SampEn) measures. Models controlled for all covariates listed in Methods.

Cognitive Measure	CV Measure (SampEn)	IRR	*p*	95% CIs	*n*
MOCA Errors W1	sBP	1.38	**<0.001**	1.25 to 1.52	4525
	dBP	1.21	**<0.001**	1.10 to 1.35	4525
MMSE Errors W1	sBP	1.40	**<0.001**	1.17 to 1.68	4525
	dBP	1.35	**0.001**	1.13 to 1.62	4525
MOCA Errors W1 and 3	sBP	1.47	**<0.001**	1.24 to 1.74	3600
	dBP	1.19	**0.047**	1.00 to 1.42	3600
MMSE Errors W1–5	sBP	1.51	**0.001**	1.17 to 1.95	4316
	dBP	1.42	**0.009**	1.09 to 1.84	4316

Abbreviations: IRR: incident rate ratio; CIs: confidence intervals.
